# Current News

**Published:** 1889-03

**Authors:** 


### Current News.

The Dental Protective Association is all the rage.

Don't forget to read their circular and send in your membership fee at once.

The International Tooth Crown Company has added new patents covering the principle involved by *removable bridge work* and other appliances in use by the profession.

"When a dental journal must remind its readers that 'it still leads,' is it not time to begin to see if some other journal is not doing something in the same line."—*Dental Review.*

Those are our sentiments, too.—Ed.]

Dr. W. N. Morrison states that deposits may be removed from very loose teeth by using a very delicate long-bladed spicula forceps, one hand resting on the incising edge of the tool to steady it.

The dental student has only time for that which is essential to dental practice. He needs dental therapeutics, dental pathology, and dental anatomy. He has no need for the anatomy of the foot, for he always stands at the head.

Hygiene, says Dr. George J. Friedrich, begins where two wandering molecules meet, and begin what you and I are to be, and last till these elements are restored to dust. We are created but little lower than the angels, but neglect of this beautiful casket is the most frequent source of sorrow and suffering.

Regarding the toxological effects of cocaine, Dr. Story relates the following: "In using cocaine for spraying the throat some was spilled on the floor, and the next morning three dead rats were found on the spot. To test the matter, some four per cent. solution was purposely spilled, and the next morning four more dead rats were found." He thinks this should make us more cautious in its use.

In his paper on "Dental Literature," read at the Louisville meeting, Dr. B. Holly Smith says the degree of D. D. S. can no longer be the badge of partial culture, but must represent the accomplishment of certain definite, specified work, requiring abundant ability, patient study and constant application; and that all recent writers, while sadly at variance with regard to the methods by which it is to be obtained, are in perfect harmony as to the advisability of it, and the belief in its ultimate adoption; all admitting, however, the necessity for a thorough preparatory education.

With regard to dental college education, Dr. Ingersoll, in his paper on the subject, says: "While lectures are an economy of time and labor to the teacher, the student must have a disciplined mind to reap the full benefit of this system. As a rule, the lecturer has a too rapid flow of ideas for the average pupil to catch, and not the full import of his remarks. The knowledge gained, as a rule, from lectures is extremely small, unless the pupil has read up reference books, in which, however, he will recognize only here and there a thought of the lecturer. Our text-books are abundant and valuable, but no time is allotted for their study, and it must be taken from sleep."

At the Louisville meeting, last September, Dr. Sudduth said he considered the prospects of success much more favorable in implantation than in transplantation. In the first place a healthy tooth is placed in a clean socket in healthy tissues. In the latter case a tooth so badly diseased that it is condemned is replaced in necrosed and abscessed territory, where everything is against it. In implantation there is no retrograde tendency to overcome. The only difficulty in the case of an implanted tooth is its fixed, immovable position without the interposed elastic cushion of pericementum, which enables the normal tooth to resist the shock of mastication.

Dr. Catching states that union by ankylosis was proven in the case of a tooth implanted by Dr. E. S. Chisholm. On its removal it was found necessary to nip off the adhering alveolar process, so firm was the union.

Dr. Ingersoll claims that dentistry, in the common acceptation of the term, is not a specialty of medicine. Professors in medical science cannot give instruction in practical dentistry. Dentistry is not a specialty of medicine either in its literature, in its colleges, or in its teachers. It is an independent science. The student of dental anatomy does not study medicine any more than the school-girl who studies a school text-book on anatomy and physiology. She also studies chemistry; but it is as a natural science, not as medicine. The clinical rooms of a dental college should not be called an infirmary. Patients do not come there because of their infirmities.

Dr. Taft considers that all the discussion of the relations of dentistry and medicine are most unprofitable. The two are so interblended and interwoven that it is utterly impossible to separate them. Dentistry is a most important branch of the healing and restorative art.

Regarding the manner of instruction in our colleges, he thinks it would be profitable to have a normal class of teachers, who could spend a week or so in discussing methods. As each finds a better method, let him give it to others. Interchange of visits would also be beneficial. A valuable feature is a syllabus of each lecture, with reference to pages of books of reference that deal tersely with certain points, and which the student is required to study up. Requiring him to write out his own views after posting himself will make the student studious and careful.

College instruction, Dr. Morgan says, should be imparted more by recitations and demonstrations than it is. Forty descriptions of a thing are not equal to one sight of it.

---

### DR. CARL SEILER'S ANTISEPTIC SPRAY FOR REDUCING ACUTE AND SUBACUTE INFLAMMATION OF NASAL MUCOUS MEMBRANE.

| | |
|---|---|
| Sodii bicarb | ʒ viij. |
| Sodii bibor | ʒ viij. |
| Sodii benzoate, | |
| Sodii salicylate | āā gr. xx. |
| Eucalyptol, | |
| Thymol | āā gr. x. |
| Menthol | gr. v. |
| Ol. gaultheria | gtt. vj. |
| Glycerine | ℥ viiiss. |
| Alcoholis | ℥ ij. |
| Aquæ | q. s. 16 pints. |

This formula gives a solution which is sufficiently alkaline to dissolve the thickened secretion adhering to the nasal mucous membrane, and as it is of the proper density, it is bland and unirritating, leaving a pleasant feeling in the nose. At the same time it is antiseptic and acts as a deodorizer, being in this respect far superior to Dobell's solution or any other non-irritating deodorizer and antiseptic. As it is, however, inconvenient for many patients to have so large a quantity of solution on hand, one of our Philadelphia druggists made the solid ingredients into a compressed tablet, so that one, when dissolved in two ounces of water, will make a solution identical in its effects with the solution made after the above formula, and my patients prefer the tablets to the solution.—*From a Reprint.*

[This solution forms a most excellent mouth wash in stomatitis and ulitis from any cause.—Ed.]

